# Sleep Is Compromised in −12° Head Down Tilt Position

**DOI:** 10.3389/fphys.2019.00397

**Published:** 2019-04-16

**Authors:** Alessa L. Boschert, David Elmenhorst, Peter Gauger, Zhili Li, Maria T. Garcia-Gutierrez, Darius Gerlach, Bernd Johannes, Jochen Zange, Andreas Bauer, Jörn Rittweger

**Affiliations:** ^1^Department of Muscle and Bone Metabolism, German Aerospace Center (DLR), Institute of Aerospace Medicine, Cologne, Germany; ^2^Forschungszentrum Jülich, Institute of Neuroscience and Medicine (INM-2), Jülich, Germany; ^3^Division of Medical Psychology, University of Bonn, Bonn, Germany; ^4^State Key Laboratory of Space Medicine Fundamentals and Application, China Astronaut Research and Training Center, Beijing, China; ^5^High Studies Center Alberta Gimenez (CESAG) University of Comillas, Palma de Mallorca, Spain; ^6^Neurological Department, Medical Faculty, Heinrich-Heine-University, Düsseldorf, Germany; ^7^Department of Pediatrics and Adolescent Medicine, University of Cologne, Cologne, Germany

**Keywords:** head down tilt, simulated microgravity, bed rest, polysomnography, sleep

## Abstract

Recent studies are elucidating the interrelation between sleep, cranial perfusion, and cerebrospinal fluid (CSF) circulation. Head down tilt (HDT) as a simulation of microgravity reduces cranial perfusion. Therefore, our aim was to assess whether HDT is affecting sleep (clinicaltrials.gov; identifier NCT 02976168). 11 male subjects were recruited for a cross-over designed study. Each subject participated in two campaigns each comprising 3 days and 2 nights. Intervention started on the second campaign day and consisted of maintenance of horizontal position or −12° HDT for 21 h. Ultrasound measurements were performed before, at the beginning and the end of intervention. Polysomnographic measurements were assessed in the second night which was either spent in horizontal posture or at −12° HDT. Endpoints were sleep efficiency, sleep onset latency, number of sleep state changes and arousals, percentages of N3, REM, light sleep stages and subjective sleep parameters. N3 and REM sleep reduced by 25.6 and 19.1 min, respectively (*P* = 0.002, *g* = -0.898; *P* = 0.035, *g* = -0.634) during −12° HDT. Light sleep (N1/2) increased by 33.0 min at −12° HDT (*P* = 0.002, *g* = 1.078). On a scale from 1 to 9 subjective sleep quality deteriorated by 1.3 points during −12° HDT (*P* = 0.047, *g* = -0.968). Ultrasonic measurement of the venous system showed a significant increase of the minimum (*P* = 0.009, *P* < 0.001) and maximum (*P* = 0.004, *P* = 0.002) cross-sectional area of the internal jugular vein at −12° HDT. The minimum cross-sectional area of the external jugular vein differed significantly between conditions over time (*P* = 0.001) whereas frontal skin tissue thickness was not significantly different between conditions (*P* = 0.077, *P* = 0.811). Data suggests venous congestion at −12° HDT. Since subjects felt comfortable with lying in −12° HDT under our experimental conditions, this posture only moderately deteriorates sleep. Obviously, the human body can almost compensate the several fold effects of gravity in HDT posture like an affected CSF circulation, airway obstruction, unusual patterns of propioception and effects on the cardiovascular system.

## Introduction

Head down tilt (HDT) reduces intracranial perfusion and leads to jugular vein congestion (Marshall-Goebel et al., 2016; Kramer et al., 2017). Moreover, an increased intracranial pressure is a consequence of HDT (Lawley et al., 2017). Xie et al. (2013) have demonstrated that sleep, among other factors, is vital for cranial fluid exchange and metabolic clearance of by-products via the “glymphatic” system. Transport via the “glymphatic” system is also dependent on body position (Lee et al., 2015). Yet one of the major drivers for intracranial fluid regulation is respiration (Dreha-Kulaczewski et al., 2015; Delaidelli and Moiraghi, 2017). Body position, altered respiration and the accompanying changed intracranial fluid dynamics are likely to affect sleep at HDT.

Of note, HDT is also used as a ground-based model of spaceflight (Kakurin et al., 1976; Pavy-Le Traon et al., 2007). Fluid shifts in the direction of the head and venous congestion in particular are salient effects of both spaceflight and HDT.

Sleep is often disturbed in astronauts. Reduced sleep quality is frequently ascribed to the “multistressor environment” onboard the space station, comprising challenges by noise, workload, lighting, sleep shifting and hypercapnia. All these factors combined eventually result in deteriorated sleep quality and quantity. A daily sleep period of a sufficient time (8.5 h) is set during missions. However, astronauts reported an actual sleep duration of only about three quarters of the available sleeping time (Dinges et al., 2013). Sleep duration is further reduced during periods of circadian misalignment (Flynn-Evans et al., 2016). The shortened and disturbed sleep during shuttle flights and on the International Space Station (Dijk et al., 2001; Whitmire et al., 2009) is changed to an extent that has detrimental effects on cumulative sleep duration and performance in Earth-based studies (Van Dongen et al., 2004). Polysomnographic recordings showed altered sleep architecture with a reduction in deep sleep (Gundel et al., 1997; Dijk et al., 2001). This is in accordance with astronauts specifically complaining about impaired refreshment post-sleep (Dijk et al., 2001) as deep sleep is vital for recovery. The reduction in sleep duration also results in increased susceptibility to stress while physical exhaustion rises due to the impaired sleep quality (Dinges et al., 2013). All these factors combined ultimately result in a higher risk of misconception and errors. Additionally, sleep disturbance is a main reason for medication intake in space (Barger et al., 2014). The effects of these medications – for example sleepiness – can last for several hours. This, again, places the astronauts at higher risk during work periods. The described reasons for inefficient sleep and their possible impact on astronauts’ performance necessitate a better understanding of sleep during microgravity as well as during ground-based bed rest studies as a simulation of microgravity.

To the best of our knowledge, it had not been considered that alterations in intracranial fluid regulation, clearance of metabolites and sleep performance can impact each other negatively at −12° HDT. However, based on the alterations above, it seems quite possible. There are two studies reporting a reduction in deep sleep (stage 4 according to Rechtschaffen and Kales) as well as increased arousal frequency during experimental −6° HDT bed rest (Mizuno et al., 2005; Komada et al., 2006) that lasted for 3 days. Both studies used a counterbalanced design with the subjects staying in horizontal position at another session. Gkivogkli et al. (2016) assessed sleep parameters repeatedly during a 50 days −6° bed rest study. Subjects were randomly assigned to two groups. In contrast to the control group, the training group performed reactive sledge jumping three to four times a week. The preliminary results published in the abstract for the SAN2016 Meeting suggest an increase in N1 sleep stage, and a decrease in the total sleep time, N2, N3, and REM sleep stage in the control group between baseline and 21 days of −6° HDT. In the training group N1 and N3 sleep stage seem to increase, N2 sleep stage decreases, while duration REM sleep stage does not change on the 21st day of −6° HDT compared to baseline. Meck et al. (2009) reported that about half of their subjects complained about impaired sleeping, especially in the first week of their 90 day head down bed rest, but also before starting the bed rest. This is in accordance with results from a study conducted by DeRoshia and Greenleaf (1993). Subjects’ subjective sleep quality improved after finishing the 30 days −6° bed rest. However, HDT-effects in the last three bed rest studies were assessed longitudinally, and may thus have been confounded with effects of isolation or lack of exercise. Yet, evaluation of objective sleep parameters is not done in most studies.

Therefore, we decided to study the short-term effects of HDT on sleep using the current guidelines of the American Academy of Sleep Medicine (AASM). As our interest was based on postulated effects of intracranial fluid regulation upon metabolic clearance, we decided to limit the exposure to HDT to one night only. We hypothesized that HDT will negatively affect sleep performance. We deliberately used −12° HDT rather than −6° HDT as −12° HDT has shown to be more efficient when measuring possible correlation between intracranial fluid systems (Marshall-Goebel et al., 2016; Kramer et al., 2017).

## Materials and Methods

### Subjects

The study has been registered at clinicaltrials.gov (identifier NCT 02976168). It conformed to the declaration of Helsinki and had been approved by the ethics committee of the regional medical board (Ärztekammer Nordrhein). All subjects gave written informed consent in accordance with the Declaration of Helsinki. The IPCog Study (Intracranial Pressure and brain function: effects of HDT upon brain perfusion and cognitive performance) was conducted at the :envihab, German Aerospace Centre (DLR), Cologne, Germany. Thirteen male subjects were recruited for the study. Volunteers’ physical and psychological health was confirmed with questionnaires and medical examination. Exclusion criteria comprised among others a history of sleeping disorders, gastro-esophageal reflux, hiatus hernia, abuse of alcohol, medication or drugs and smoking within a period of 6 months prior to the study (Table 1). One week prior to the study onwards, subjects did not consume any caffeine. Two out of the thirteen subjects dropped out after their inclusion. One had to leave during data collection because of family issues, and the other reported gastro-esophageal reflux and vomiting during the −12° HDT night. This made it impossible to stay in HDT position. The 11 subjects finishing the study had a age of 30 years ± 10.2 years, a height of 179.82 cm ± 6.6 cm and a weight of 79.82 kg ± 7.3 kg, mean ± SD.

**Table 1:** Exclusion criteria.

Exclusion criteria of the IPCog study	
Medical history	Migraine/chronic headaches Psychological /central nervous disorders Sleeping disorders/inability to sleep on the back Diabetes mellitus Pronounced orthostatic intolerance Kidney disorder Thyroid gland disorder Anemia Lumbar surgery/lumbar spine trauma/ chronic back complaints Elevated risk of thrombosis or coagulopathy Hiatus hernia/gastro-oesophageal reflux Ophthalmological conditions, such as glaucoma etc. weak concentration/previous psychiatric illness
	
Medication/Substance intake	Drug, medication, alcohol abuse Smoking within 6 months prior to study Medication impairing cognitive function, autonomic function Medication influencing any of the study procedures
	
Others	Motor/sensory deficits Contraindications against MRI Any medical condition considered a contraindication to study procedures/would make it unsafe/confound measurements

### Study Design and Conduction

**FIGURE 1:**
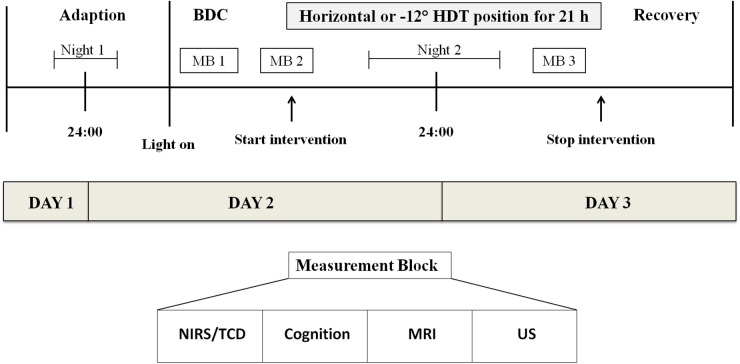
Campaign overview. Each subject participated in two campaigns, once staying in horizontal posture and once being tilted to −12° HDT. Between these two campaigns there was about a week’s time. Measurement blocks were carried out on both interventional days comprising five measurements. BDC, Baseline data collection; MB, Measurement block; NIRS, Near-infrared spectroscopy; TCD, Transcranial Doppler ultrasound of the mid-cerebral artery; CTB, cognitive test battery; MRI, (functional) magnetic resonance imaging; US, ultrasound of the jugular vein and facial thickness.

Polysomnographic recordings were one of the secondary outcome measures of the IPCog study, with the aim to substantiate any detrimental effects of sleeping in −12° HDT position. Primary outcome was the result of the cognitive test battery. The study was carried out in a cross-over design from March 2016 to November 2016, totaling 10 campaigns. Each campaign consisted of 3 days and, therefore, two nights (Figure 1). The interventional phase started on the second campaign day, lasting for approximately 21 h. Subjects spent the whole interventional phase either lying in horizontal position or at −12° HDT.

During the first night subjects slept in horizontal posture. The second night belonged to the interventional period. Therefore, subjects slept either at −12° HDT as experimental condition or horizontally as control condition. All subjects participated in both campaigns with approximately a week’s time period in between.

Subjects participated in groups of three or two subjects. Each of them slept in their own room which was the same for both campaigns. To maintain −12° HDT and 0°, respectively, subjects did not use pillows.

The polysomnographic device was applied during both nights of each campaign. Lights on and off times were fixed beforehand and arranged stepwise for the subjects participating in the same campaign (21:45–06:00, 22:00–06:30, 22:30–07:15). Stepwise arrangement and the minor difference in lights-off time were due to the arrangement of daytime measurements. Otherwise it would not have been possible to perform two measurement blocks on the second campaign day. To record any aberration from the predefined sleeping times, subjects were asked to set a marker when the light was turned off. Subjects were not allowed to sleep outside those predefined sleeping times.

While sleeping at −12° HDT, special mattress pads were used to prevent sliding headwards. Belts were provided to facilitate the subjects pulling themselves up toward feet direction. An additional belt was provided offering the subjects the possibility to fix their feet and, thus, prevent sliding. This was done to minimize any effects on sleeping due to sliding. Additionally, subjects suffering from back pain during −12° HDT were offered a painkiller (Ibuprofen 400 mg) upon request and under medical supervision. Out of all, only subject 0E and 0F were given one tablet each before going to sleep at −12° HDT.

Whether subjects stayed in horizontal or −12° HDT position first was randomized directly before the start of the interventional phase in the afternoon of the campaign’s second day. Randomization was done according to even and uneven numbers of a dice throw. To ensure a balanced assignment a maximum two out of three subjects participating in the same campaign could potentially start at −12° HDT. If the group comprised of two subjects, only one of them was tilted to −12° HDT during the first campaign. Randomization was performed by scientific staff members.

Six of the eleven subjects were tilted to −12° HDT during the first campaign and remained in horizontal posture during the second. Five of them started in horizontal position during the first campaign and stayed at −12° HDT during the second campaign.

### Data Acquisition

Polysomnographic data were recorded with two portable SOMNOscreen Plus devices (SOMNOmedics, Randersacker, Germany) and with one SOMNOscreen device (SOMNOmedics, Randersacker, Germany). Subjects were tested with the same device type during both interventional phases. Recordings included electroencephalography (EEG), electrooculography (EOG), electrocardiography (ECG), submental electromyography (EMG), peripheral oxygen saturation at finger level, as well as abdominal and thoracic belts to assess breathing movement. With the SOMNOscreen Plus devices, EEG electrodes were placed at positions O1, O2, C3, C4, F3, and F4 according to the international 10–20 System and at O1, C3, C4, and F3 positions with the SOMNOscreen device. Additional electrodes were placed at the mastoids on both sides, ground and reference position in all devices. Cup electrodes by Ambu (Ambu Neuroline Cup, Bad Nauheim, Germany) were used and fixed with EC2 electrode cream by Grass. To further ensure sufficient adhesion of the electrodes small pieces of compresses (Gazin Mullkompressen, Lohmann and Rauscher, Rengsdorf/Neuwied, Germany) were used to fixate the electrodes. For the submental derivations and on other positions where applicable, adhesive electrodes by Ambu (Ambu Neuroline 720, Bad Nauheim, Germany) were used. During the entire study, electrodes were positioned by the same operator. Before attaching the electrodes the skin was prepared with Nuprep skin Prep Gel (Weaver and Company, Aurora, United States) and cleaned with alcoholic disinfectant. This was done to lower skin impedance in order to improve EEG signal strength. An impedance of less than 10 kΩ (calibrated against the mastoid electrodes) was considered to be sufficient.

During the cognitive test battery conducted on the morning of the second and third day subjective sleep quality and subjective refreshment were assessed by using a scale from one to nine. These scales were devised by the scientific team. In both scales, nine represented the best quality of sleeping and resting, respectively. The subjects also indicated their sleep duration by stating their assumed sleeping hours.

Effects of body position on the venous system were assessed during measurement blocks on the second and third campaign day. Frontal tissues thickness was obtained as a surrogate parameter for potential fluid shifts toward the head in general. Measurements were always in the second time slot of each measurement block. Therefore, measurements during the first and third measurement block were performed at the same time of the day. This allowed accounting for potential daytime-dependent effects when comparing measurements of the same campaign.

Data was obtained by B-mode ultrasound using a myLab 25 scanner with a linear 8–10 MHz probe (Esaote, Milan, Italy). The same operator performed the measurement throughout the study. Tissue thickness was assessed on both sides of the front. For each side three measurements during each measurement block were performed. Minimum and maximum cross-sectional areas of the internal and external jugular veins were assessed on both sides. The probe positions were marked on the subject’s skin in measurement block 1 for subsequent measurements of the campaign.

Assessment of cranial perfusion included transcranial Doppler ultrasound (TCD), near infrared spectroscopy (NIRS), MRI measurements and a cognitive test battery. TCD and NIRS measurements were assessed simultaneously. Each assessment lasted for at 20 min. Measurements during the first measurement block also included the tilting process: NIRS and TCD assessment was performed for 20 min in horizontal position. The subjects were the tilted during the measurement and data acquisition then continued for further 20 min. MRI measurements were carried out using a special wedge to maintain −12° HDT position. MRI sequences comprised of functional MRI [echo planar imaging – blood oxygenation level dependent (BOLD)], T1 weighed magnetization prepared rapid gradient echo (MPRAGE), pulsed arterial spin level (pASL) with an inversion time (TI) of 1800 ms and phase contrast (PC) imaging. A cognitive test battery was used to assess executive functions, psychomotor vigilance, reaction time, logic, working memory and continuous performance. Analysis is currently ongoing.

### Data Processing and Analysis

The manual EEG analysis met the criteria of the AASM. After having applied an ECG filter, each night’s sleep was analyzed in intervals of 30 s classifying them as either sleep stages N1–N3 and REM or awake according to the rules of the AASM (Iber et al., 2007). Additionally, we assessed sleep onset latency, changes of sleep states and sleep efficiency (percentage of sleeping time of in bed time). We used the DOMINO Software (Version 2.7.0, 07.09.2015) by SOMNOmedics (SOMNOmedics, Randersacker, Germany). The channels used were EOG, EEG and submental EMG. Each of the EEG signals was derivated against the signal of the mastoid (M1, M2) electrodes on the opposite side (O1:M2, O2:M1, C3:M2, C4:M1, F3:M2, F4:M1). Where possible, the EEG signals from the left side were used for analysis. Therefore the electrode positions O1, C3, C4, and F3 were used with the SOMNOscreen device. AASM criteria for arousals were applied.

Total sleep time was used, rather than the time in bed, to prevent confounding influences by lights on/off periods. The polysomnographies were all analyzed by the same operator to avoid any interpersonal difference in the analysis. The operator was a scientific staff member of the study and, therefore, not blinded.

Analysis of peripheral tissue oxygenation at the index or middle finger included the mean oxygenation, minimum oxygenation and the time peripheral tissue oxygenation was below 90% as given by the DOMINO Software after the automatic exclusion of artifacts. Abdominal and thoracic belts often shifted out of place due to the subjects’ sliding toward head direction. Therefore, an adequate analysis of breathing movement was not possible.

Whilst the polysomnographic devices were applied during the first night for habituation purposes they were not analyzed. This was done to reduce any influences of the first-night effect on the actual experimental night. Therefore, only the second night was statistically analyzed. Likewise, subjective sleep quality was evaluated solely for the experimental night.

The mean of the three measurements performed during one session on one side was used for analysis of frontal tissue thickness. Variation in jugular vein diameter was determined by substracting the minimum diameter of each vein from its maximum diameter.

Due to technical issues, ultrasonic measurements of subject R and S could not be performed. Therefore, they were not included in the statistical analysis.

Data analysis was based on the per protocol approach, therefore excluding the two drop-outs mentioned beforehand. Defining the experimental condition (0° or −12° HDT) as fixed effect and subjects as random effects, linear mixed models were applied. Additionally, the models were checked for non-normal distribution of residuals and heteroscedacity. Box-cox transformations were used when needed. The level of significance was set at *P* = 0.05. R Studio (Version 1.0.136, R Studio) was used to carry out the statistical analysis, hereby using the lme-function to create the linear mixed models. Because of the small sample size Hedge’s g was used to calculate the effect size rather than Cohen’s d.

## Results

A significant increase (*P* = 0.002, *g* = 1.078) of the percentage of light sleep (N1 + N2) of the total sleep time could be detected during −12° HDT (59.9% ± 7.2%) in comparison with 0° (50.9% ± 9.4%). The fraction increased in 10 out of the 11 subjects, the maximum rise being 21.6%. One of the subjects (0J) showed a slight decrease of the light sleep (-3.1%) during the −12° HDT night.

There was a significant decrease in percentage of slow wave sleep (N3) (*P* = 0.002, *g* = -0.898) from the 0° night (25.4% ± 6.4%) to the −12° HDT night (20.2% ± 5.2%). This decrease ranged between 1.4 and 12.7% in 10 subjects, while the amount of N3 remained the same (17.9%) in the last subject (0E) during both nights.

**FIGURE 2:**
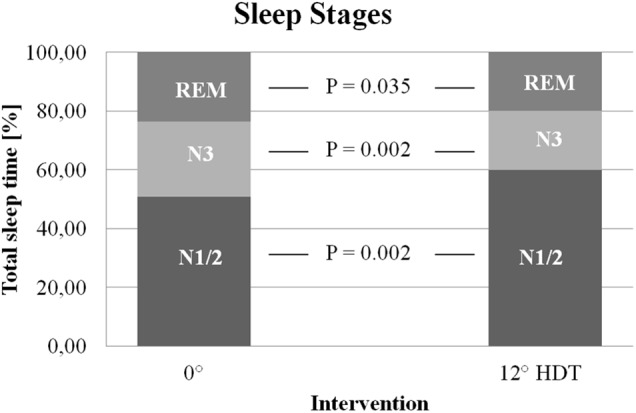
Sleep stages. Percentage of slow wave sleep (20.2% ± 5.2% vs. 25. 4% ± 6.4%) and REM sleep (19.9% ± 5.7% vs. 23.7% ± 6.3%) were reduced during the −12° HDT night. Percentage of light sleep increased (59.9% ± 7.2% vs. 50.9% ± 9.4%) when compared to horizontal position. HDT, Head down tilt.

There was a significant decrease (*P* = 0.035, *g* = -0.634) in the percentage of REM sleep of the total sleep time when comparing the night in horizontal position (23.7% ± 6.3%) to −12° HDT (19.9% ± 5.7%). The extent of the decline varied between 1.5 and 9.7%. 2 Subjects (0J, 0N) showed an increase in the amount of REM sleep during −12° HDT (Figure 2).

The mean time period for each of the sleep stages can be seen in Table 2. There were no significant differences between 0° position and −12° HDT position.

**Table 2:** Objective sleep parameters.

	0° position		−12° HDT position	
TST (min)	422.00 ± 79.10		410.40 ± 55.00	
Sleep onset latency (min)	24.90 ± 29.10		35.00 ± 13.70	
Number of sleep state changes	119.00 ± 33.20		128.10 ± 37.30	
Sleep efficiency (%)	82.40 ± 14.70		80.10 ± 9.60	

**Sleep stages**	**(min)**	**(% of TST)**	**(min)**	**(% of TST)**

Light sleep (Stage N1/N2)	211.53 ± 45.50	50.9 ± 9.4	244.49 ± 34.10	59.9 ± 7.2^∗^
Slow wave sleep (Stage N3)	107.86 ± 34.77	25.4 ± 6.4	82.23 ± 22.59	20.2 ± 5.2^∗^
REM sleep	102.77 ± 36.37	23.7 ± 6.3	83.64 ± 31.49	19.9 ± 5.7^†^

*Table giving an overview of the objective sleep parameters at −12° HDT and horizontal position. The values are given in means ± standard deviation. ^∗^P = 0.002, ^†^P = 0.035 in comparison to (% of TST) in 0° position. HDT, Head down tilt; TST, Total sleep time. Sleep efficiency was not significantly different (P = 0.48) between the −12° HDT night (80.1% ± 9.6%) compared to that spend in 0° (82.4% ± 14.7%).*

Total sleep time was not significantly affected (*P* = 0.44) by −12° HDT (410.4 min ± 55 min vs. 422 min ± 79.1 min at 0°). There was no significant difference in sleep onset latency (*P* = 0.30). Both, maximum (110 min) and minimum (2.0 min) onset latency occurred during the night subjects stayed in horizontal posture. However, when excluding the subject with the maximum sleep onset latency, the latency is significantly prolonged at −12° HDT. We found no significant difference in the number of sleep state changes when comparing the two experimental conditions (−12°: 128.1 ± 37.3; 0°: 119 ± 33.2; *P* = 0.19).

The mean peripheral tissue oxygenation at finger level remained unchanged (94.5%) during both nights with standard variation only differing slightly (2.8% vs. 2.9%). The lowest basal oxygenation of 88% was measured during −12° HDT. There was no significant change in minimum oxygenation detectable (−12°: 85.9% ± 6.8%; 0°:83.6% ± 11%; *P* = 0.41). The percentage of the mean time with the peripheral tissue oxygenation being lower than 90% showed no significant difference (*P* = 0.95) (Table 3).

**Table 3:** Measurement of peripheral oxygen saturation at finger level.

	0°	−12° HDT
Mean SpO_2_ (%)	94.50 ± 2.90	94.50 ± 2.80
Minimal SpO_2_ (%)	85.90 ± 6.80	83.60 ± 11.00
SpO_2_ < 90% (%) [(min)]	10.70 ± 22.00 (31.29 ± 83.49)	11.30 ± 21.90 (40.71 ± 89.81)

*Mean SpO_2_, minimal SpO_2_ and the amount of time with an SpO_2_ < 90% were assessed. Subject R was excluded during the −12° HDT night because of artifacts. None of those parameters was significantly different between both conditions. The values are given in means ± standard deviation. HDT, head down tilt; SpO_2_, Peripheral oxygen saturation at finger level.*

There was a significant decrease (*P* = 0.047, *g* = −0.968) in subjective sleep quality during the −12° HDT night (4.8 ± 1.5) when compared to the 0° night (6.1 ± 1.1). However, subjective sleep duration did not differ significantly between the experimental conditions (−12°: 5.3 ± 1.8 h; 0°: 6.4 ± 1.4 h; *P* = 0.10). The subjective feeling of refreshment showed no significant difference (*P* = 0.09) between the −12° HDT night (5.6 ± 1.8) and the night spend in horizontal position (6.3 ± 1.3) (Table 4).

**Table 4:** Subjective sleep parameters.

	0° position	−12° HDT position
Subjective sleep quality (1–9)	6.1 ± 1.1	4.8 ± 1.5^∗^
Subjective refreshment (1–9)	6.3 ± 1.8	5.6 ± 1.8
Subjective sleep duration (h)	6.4 ± 1.4	5.3 ± 1.8

*Subjective sleep parameters were assessed in the morning. Sleep quality and refreshment were assessed by scales from 1 to 9. 9 represented the highest possible sleep quality and refreshment possible. Subjective amount of sleeping hours were given by the subjects. The values are given in means ± standard deviation. ^∗^P = 0.047. HDT, head down tilt.*

In comparison to the baseline data collected during measurement block 1 of each campaign frontal tissue thickness did not differ significantly on the first (*P* = 0.077) and the second interventional day (*P* = 0.811) at −12° HDT (Table 5). Frontal tissue thickness of the left and right side were differing (*P* = 0.009). There was no difference between conditions over time (*P* = 0.116).

**Table 5:** Jugular vein diameters and frontal tissue thickness.

	Baseline data collection		Measurement block 2		Measurement block 3	
	0° visit	−12° visit	0° visit	−12° visit	0° visit	−12° visit
**Internal Jugular Vein**						
Minimum cross-sectional area (cm^2^)	0.57 ± 0.42	0.65 ± 0.52	0.70 ± 0.49	1.31 ± 0.64^††^	0.54 ± 0.45	1.18 ± 0.62^†††^
Maximum cross-sectional area (cm^2^)	0.84 ± 0.49	0.87 ± 0.54	0.86 ± 0.60	1.46 ± 0.66^††^	0.76 ± 0.51	1.41 ± 0.66^††^
Variation (cm^2^)	0.27 ± 0.26	0.21 ± 0.21	0.16 ± 0.17	0.15 ± 0.10	0.22 ± 0.19	0.23 ± 0.19
**External Jugular Vein**						
Minimum cross-sectional area (cm^2^)	0.15 ± 0.07	0.15 ± 0.07	0.15 ± 0.08	0.20 ± 0.15^††^	0.15 ± 0.09	0.18 ± 0.12^††^
Maximum cross-sectional area (cm^2^)	0.17 ± 0.08	0.17 ± 0.09	0.16 ± 0.09	0.22 ± 0.16	0.16 ± 0.09	0.20 ± 0.13
Variation (cm^2^)	0.019 ± 0.027	0.025 ± 0.022	0.012 ± 0.019	0.015 ± 0.022	0.012 ± 0.010	0.018 ± 0.018
**Frontal skin thickness (mm)**	4.11 ± 0.95	4.04 ± 0.88	4.12 ± 1.01	4.26 ± 1.11	4.03 ± 0.88	4.10 ± 0.94

*B-mode ultrasound was used to assess the minimum and maximum diameters of external and internal jugular veins on both sides. Frontal tissue thickness was measured on both sides of the front. Baseline data collection in both groups was performed in horizontal posture. The values are given in means ± standard deviation. ^††^P < 0.01, ^†††^P < 0.001 for interaction between measurement block and visit.*

The maximum cross-sectional area of the internal jugular vein increased significantly at −12° HDT on interventional day 1 (1.46 cm^2^ ± 0.66 cm^2^, *P* = 0.004) and day 2 (−12° HDT: 1.41 cm^2^ ± 0.66 cm^2^, *P* = 0.002) when compared to the baseline data collection (0.87 cm^2^ ± 0.54 cm^2^). No difference could be detected when comparing the left and right side (*P* = 0.161). The minimal cross-sectional area of the internal jugular vein was also significantly different between interventional condition (−12° HDT day1: 1.31 cm^2^ ± 0.64 cm^2^, day 2: 1.18 cm^2^ ± 0.62 cm^2^) and baseline data (0.65 cm^2^ ± 0.52 cm^2^) on both interventional days (*P* = 0.009, *P* < 0.001, respectively). It did not differ between both sides over time (*P* = 0.369). The variation of the cross-sectional area of the internal jugular vein was different when comparing both sides (*P* = 0.003). It did not differ significantly between both conditions (*P* = 0.686).

The maximum cross-sectional of the external jugular vein was not significantly different with condition over time (*P* = 0.415), between the HDT- and control group (*P* = 0.128) and between sides (*P* = 0.271). However, the minimal cross-sectional area of the external jugular vein did differ between the conditions over time (*P* = 0.001). There was no significant difference between both sides over time (*P* = 0.980).

## Discussion

The percentage of slow wave sleep (N3) and REM sleep significantly decreased during −12° HDT, thereby resulting in a compensating significant increase of light sleep (N1/2). The subjective sleep quality was significantly lower when sleeping in HDT. These results are consistent with recent findings (Mizuno et al., 2005; Komada et al., 2006).

Despite these changes in sleep, the respective percentages of the sleep stages still are within the normal range for young adults (Carskadon and Dement, 2005) – although at the ends. This might be due to the subjects’ ages ranging between 21 and 55 years. This and the fact that the number of sleep stage changes remained similar during both positions suggest normal sleep architecture. Mizuno et al. (2005) drew the same conclusion at −6° HDT.

Minimum and maximum cross-sectional area of the internal jugular vein increased significantly at −12° HDT. The minimum cross-sectional area of the external jugular vein was significantly larger at −12° HDT compared to horizontal posture. Both findings indicate venous congestion at −12° HDT.

Frontal skin tissue thickness did not increase significantly during both interventional days at −12° HDT.

We had hypothesized that HDT induced changes on cerebral hemodynamics (Marshall-Goebel et al., 2016) would affect metabolic clearance and sleep. These changes in sleep might influence the transport capability of the “glymphatic” as this transport predominantly takes place during sleep (Xie et al., 2013) resulting in a reciprocal relation.

Dreha-Kulaczewski et al. (2017) showed that venous flow is a major driver for cerebrospinal fluid (CSF) circulation with both systems having opposed flow directions. Therefore, venous congestion as assessed in our study most likely results in a decreased flow of CSF into the cranium. This might directly affect metabolic clearance via CSF circulation.

Although we were not able to analyze the data of the thoracic and abdominal belts, respiration is likely to be affected by HDT: Negative thoracic pressure during inspiration is a main driver for CSF circulation (Dreha-Kulaczewski et al., 2015) as it results in a venous flow out of the cranium. Thereby, CSF flows toward the cranium. Therefore, a potential underlying cause of the increased venous congestion we observed is a change in respiration.

Respiratory obstruction may play a role in sleeping at −12° HDT. Resistance in the upper airway is associated with the lung volume (Begle et al., 1990; Stanchina et al., 2003). During HDT, lung volume decreases due to the shift of abdominal organs toward the head. This possibly results in an increased airflow resistance. The data of the abdominal and thoracic belts could not be evaluated efficiently and no difference was detected for continuous measurements of peripheral oxygen saturation. Therefore, a definite conclusion cannot be drawn.

The resulting effects of the decrease in cranial perfusion (Marshall-Goebel et al., 2016) and increased venous congestion at HDT might be similar albeit not identical to those in chronic heart failure. Chronic heart failure has a strong correlation to the obstructive sleep apnea syndrome. Although the reason for this is multifactorial one cause is possibly the fluid increase in the pharynx (Bradley and Floras, 2003).

This might also be one cause for impaired sleeping at HDT. Due to this shift, fluid also accumulates in the upper airways. The subjects were young, healthy males and the obstruction might not be as severe as in patients suffering from chronic heart failure. Thus, the full disease pattern of obstructive sleep apnea syndrome is not displayed.

In patients suffering from chronic heart failure, the retention of CSF within the cranial cavity might be the cause for cognitive impairment similar to that of idiopathic intracranial hypertension patients (Caplan, 2006). Assumed pathophysiologies for idiopathic intracranial hypertension include an increased outflow resistance of CSF (McGeeney and Friedman, 2014). Additionally, sleep apnea syndrome is related to idiopathic intracranial hypertension (Biousse et al., 2012; McGeeney and Friedman, 2014). Yet, it remains unclear whether it may be one of the causes or the result of the disease. The interactions of arterial, venous and CSF systems described above in addition with respiratory changes might be a possible approach.

With regards to obstruction, the individual build has to be taken into account. Fat distribution is an important risk factor for obstructive sleep apnea syndrome (Horner et al., 1989; Shinohara et al., 1997). Yuan et al. could show a correlation between neck circumference and pharynx collapsibility (Yuan et al., 2013) in adolescents. All our subjects’ BMIs were within the normal range but minor influences of individual body fat distribution cannot be completely ruled out.

The change of sleep stages observed in our study could also be explained by a raised level of physiological stress hormones during HDT as Norepinephrine is one of the transmitters influencing REM sleep stage (McCarley and Hobson, 1975; Winokur et al., 2001). Although, Rai and Kaur (2011). found that said increase in stress hormones took place only after the first week of bed rest, subjects were only positioned at −6° HDT. −12° HDT might lead to a quicker response of the stress system.

Mood, especially an increase in depression, plays a major role during HDT bed rest studies (Styf et al., 2001; Pavy-Le Traon et al., 2007) – even though not always reaching clinically significant level (Liu et al., 2012). As depression and sleep influence each other mutually (Kaneita et al., 2006), this has to be taken into account. However, −12° HDT was applied for less than 24 h and depressive disorders were an exclusion criterion. Liu et al. found no significant changes in anxiety level at −6° HDT (Liu et al., 2012) rendering altered mood an unlikely confounder of sleep (Rosa et al., 1983).

Gundel et al. observed a change in circadian rhythm of astronauts (Gundel et al., 1997) assessed by measuring the body temperature. During bed rest studies these changes generally occur at a later time (Pavy-Le Traon et al., 2007). Therefore, this factor did most likely not affect sleep in our study.

Whereas the subjective feeling of refreshment and estimated sleep duration were not influenced by the body position, subjective sleep quality was significant lower at −12° HDT which might be explained by the significant reduction of slow wave sleep (N3). Both slow wave sleep and sleep efficiency affect subjective sleep quality (Keklund and Akerstedt, 1997). Yet, when comparing subjective with objective sleep quality the results remain inconsistent (Akerstedt et al., 1994).

One limitation of our study regarding sleep was the issue of sliding at −12° HDT. Despite applying several countermeasures, subjects still described wakening because of reaching the head-end of the bed. This impedes the distinction between the effects of HDT itself and those of the sliding on the sleep behavior. The issue of sliding might also have contributed to deterioration in subjective comfort.

The study was designed to diminish any impact of the first night effect. A recent finding indicates REM sleep might even be influenced up to four nights (Le Bon et al., 2001). This might have affected our measurements. However, the counterbalanced design of the study should have reduced the possible impact on sleep.

Before sleeping, two subjects were given painkillers. Although none of the subjects woke up due to pain, the general increase in back pain, stomach pain and headache during bed rest (Styf et al., 2001) might still have influenced their sleeping. Apart from that, several subjects described a feeling of distention in their upper abdomen during −12° HDT contributing to general discomfort. Ibuprofen could affect sleep (Murphy et al., 1994) via inhibition of prostaglandin synthesis and the consequential change in body temperature and melatonin synthesis (Murphy et al., 1996). Yet, these results could not be confirmed (Gengo, 2006).

As with every bed rest study, there remains the question to what extent results are valid for actual space missions. With the observations being generally applicable this comparison might still be impeded due to surrounding circumstances during spaceflight, such as noise and mission parameters (Monk et al., 1998; Mallis and DeRoshia, 2005).

Overall, we could confirm previous findings from bed rest studies regarding sleep stage changes and sleep architecture. We also hypothesized that interrelations between the cranial perfusion systems and metabolic clearance would be a possible explanation for the poorer sleep behavior at −12° HDT. All in all, a better understanding of sleep and its relation to the intracranial fluid system is not only relevant for future space missions but might also have implications for clinical medicine.

## Ethics Statement

This study was carried out in accordance with recommendations of the Declaration of Helsinki in its latest version, Datenschutzgesetz (Data Protective Act), Medizinproduktgesetz (MPG) (Medical Device Law), and Medizinprodukte – Betreiber Verordnung (MPBetrV). The protocol was approved by the ethics committee of the regional medical board (Ärztekammer Nordrhein). All subjects gave written informed consent in accordance with the Declaration of Helsinki.

## Author Contributions

All authors contributed to the hypothesis development, study design, data acquisition, and manuscript finalization. Analysis of the IPCog study results were carried out by ALB, ZL, DG, BJ, JZ, and JR. PG and JR supervised the study. ALB wrote the first draft of the manuscript and designed the graphs.

## Conflict of Interest Statement

The authors declare that the research was conducted in the absence of any commercial or financial relationships that could be construed as a potential conflict of interest.

## Abbreviations

HDT: Head down tilt
NIRS: Near infrared spectroscopy
TCD: Transcranial Doppler ultrasound.
